# Direct and indirect effects of retinoic acid on human Th2 cytokine and chemokine expression by human T lymphocytes

**DOI:** 10.1186/1471-2172-7-27

**Published:** 2006-11-21

**Authors:** Harry D Dawson, Gary Collins, Robert Pyle, Michael Key, Ashani Weeraratna, Vishwa Deep-Dixit, Celeste N Nadal, Dennis D Taub

**Affiliations:** 1 Nutrient Requirement and Functions Laboratory, United States Department of Agriculture, Beltsville, MD, USA; 2 Laboratory of Immunology, Gerontology Research Center, National Institute on Aging, NIH, Baltimore, MD, USA

## Abstract

**Background:**

Vitamin A (VA) deficiency induces a type 1 cytokine response and exogenously provided retinoids can induce a type 2 cytokine response both in *vitro *and *in vivo*. The precise mechanism(s) involved in this phenotypic switch are inconsistent and have been poorly characterized in humans. In an effort to determine if retinoids are capable of inducing Th2 cytokine responses in human T cell cultures, we stimulated human PBMCs with immobilized anti-CD3 mAb in the presence or absence of all-*trans *retinoic acid (ATRA) or 9-*cis*-RA.

**Results:**

Stimulation of human PBMCs and purified T cells with ATRA and 9-cis-RA increased mRNA and protein levels of IL-4, IL-5, and IL-13 and decreased levels of IFN-γ, IL-2, IL-12p70 and TNF-α upon activation with anti-CD3 and/or anti-CD28 mAbs. These effects were dose-dependent and evident as early as 12 hr post stimulation. Real time RT-PCR analysis revealed a dampened expression of the Th1-associated gene, T-bet, and a time-dependent increase in the mRNA for the Th2-associated genes, GATA-3, c-MAF and STAT6, upon treatment with ATRA. Besides Th1 and Th2 cytokines, a number of additional proinflammatory and regulatory cytokines including several chemokines were also differentially regulated by ATRA treatment.

**Conclusion:**

These data provide strong evidence for multiple inductive roles for retinoids in the development of human type-2 cytokine responses.

## Background

An uncommitted precursor T helper (pTh) cell can be induced to differentiate into at least two distinct subsets of effector cells, T helper type 1 (Th1) and T helper type 2 (Th2) cells [1,2]. Th1 cells secrete IFN-γ, TNF-α, and TNF-β and are important for the development of delayed type hypersensitivity (DTH) reactions and protective responses to intracellular pathogens [1,2]. These cells also contribute to the pathology of autoimmune disease and graft rejection. Th2 cells express and secrete IL-4, IL-5, and/or IL-13 and are essential for the development of humoral and allergic reactions [1,2]. During T cell activation, the relative cytokine milieu within the local microenvironment is a major determinant of the direction of pTh cell differentiation. Cytokines such as IL-12 and to a lesser extent IFN-γ directly induce pTh cell differentiation into type 1 cells [1]. In contrast, IL-4 stimulates pTh cell differentiation into type 2 cells even in the presence of moderate levels of IL-12 and IFN-γ [2]. In addition to IFN-γ, IL-12 and IL-4, recent evidence also suggests an important role for cytokines such as IFN-(, IL-1α/β, IL-15, and IL-18 in stimulating type 1 responses [2] and IL-10 and IL-13 in stimulating type 2 responses [1,3]. Additional factors including hormones, growth factors and co-stimulatory molecules have also been shown to influence T cell development of type 1 or type 2 responses [1].

Vitamin A (VA) or VA-like analogs known as retinoids, are potent hormonal modifiers of rodent type 1 or type 2 responses but a definitive description of their mechanism(s) of action is lacking [4-16]. Several early studies using models of pathogen-challenged rodents indicated that VA deficiency induced a dominant Th1 response that interfered with the development of a protective humoral response [17]. These researchers proposed several potential mechanisms to account for these observations including the direct downregulation of T cell IFN-γ synthesis, direct promotion of Th2-cell differentiation, and/or alteration of accessory or antigen presenting cell function toward a Th2-inducing phenotype [18]. Recent evidence from interventional studies show that VA supplementation of VA-deficient infants and children reduces morbidity and/or mortality from measles, malaria, and certain forms of diarrhea [16]. These studies have stimulated renewed interest in elucidating VA's role in the immune response, particularly in modification of human Th1 or Th2 response development.

There are a number of contradictory findings in the literature examining the effects of retinoids on type 1 and 2 cytokine production in rodent models and cells. Several reports using murine and rat models of VA deficiency have demonstrated diminished type 1 reactions including DTH and anti-viral responses [7,19-21]. Exogenous administration of VA or RA have also been shown to increase DTH reactions and augment immune responses to viruses suggesting that these compounds potentiate type 1 reactions [21,22]. Several additional published studies using *in vitro *and *in vivo *systems of VA or retinoid deficiency and rodents and humans have also demonstrated either inhibitory, stimulatory or no effects on IFN-γ production [4-15,23,24]. As for Th2 cytokines, to date only one study has described a decrease in IL-4 production in VA deficiency [18], while several recent studies have demonstrated that retinoids induce IL-4 synthesis during *in vitro *murine T cell activation [9,25,26]. The majority of rodent studies have failed to demonstrate any effect of exogenous retinoids on IL-4 production but have observed a type 2-promoting effect of RA only when exogenous IL-4 was added to the cultures [12,22,27-29]. However, a recent study by Iwata et al. [29] demonstrated the direct effects of ATRA and 9-cis-RA on Th2 cytokine production by murine T cells derived from TCR transgenic mice. These authors also demonstrate the ability of RA to inhibit Th1 cytokine responses, while enhancing IL-4 production by Th2-polarized cells. In contrast, RA has also been shown to inhibit IL-4 production by a antigen-stimulated rat mast cell line [30] and inhibited IL-4-induced IgE synthesis from CD40-stimulated B cells [31,32]. Moreover, additional studies have suggested that VA or retinoids possess limited direct type 2 differentiating effects on purified T cells but appear to act primarily at the level of the APC by reducing type 1 cytokine synthesis [28,33]. Obviously, given all the variations in these findings, the selective differentiating effects of VA and retinoids on cytokine synthesis by T cells remains controversial. Despite all of this work, little to no detailed data exists utilizing purified human T cells and T cell subsets in such studies. In the current manuscript, we provide the first systematic analysis of the effects of the retinoids, ATRA and 9-*cis*-RA, on the development of a Th2 cytokine response as well as several cytokines and chemokines using an *in vitro *model of human T cell activation.

## Results

### ATRA and 9-cis-RA inhibit Th1 and promote Th2 reactions by human T cells and peripheral blood mononuclear cells (PBMC)

**Figure 1:**
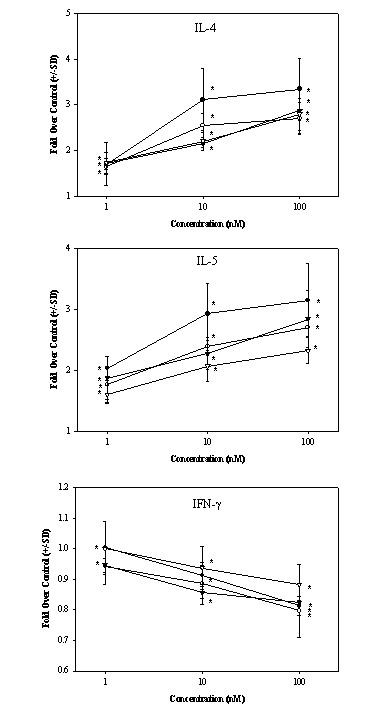
**ATRA and 9-cis-RA induces IL-4 and IL-5 and inhibits IFN-γ expression by anti-CD3 mAb-stimulated human T cells and PBMC**. IL-4, IL-5 and IFN-(levels were quantitated by ELISA in the culture supernatants of anti-CD3 mAb (200 ng/ml)-activated human T cells [circles} or PBMC [inverted triangles] (2.5 × 10^6^/ml) treated with either ATRA (filled symbol) or 9-cis-RA (open symbol). The results are expressed as fold change over vehicle control treated cells. The results shown here are the pooled data from 4 separate experiments using different PBMC donors. It should also be noted that the vehicle controls did not alter the expression of any of the cytokines in these cultures. A paired T test was performed on the combined values from each donor as described in the *Methods*. *P < 0.05.

Given the multiple reports describing the effects of retinoids on cytokine production by murine splenocytes and T cells, we initially examined the effects of various concentrations of ATRA and 9-cis-RA on the production of Th1- and Th2-associated cytokines by anti-CD3-stimulated human T cells and PBMC in vitro. Culture supernatants were examined 48 hrs after stimulation for cytokine levels. The results in Figure 1 demonstrate a representative dose response curve of ATRA and 9-cis-RA on the expression of IL-4, IL-5 and IFN-γ by human T cells and PBMC. These data reveal that the Th2 cytokines, IL-4 and IL-5, are induced in T cells and PBMC in a dose-dependent manner, while the production of the Th1 cytokine, IFN-γ, is inhibited in response to increasing concentrations of ATRA and 9-cis-RA. These findings were highly reproducible in greater than 95% of the PBMC and T cells donors examined (n > 20). Based on the above curves, ATRA and 9-cis-RA were utilized in subsequent experiments at the 10^-7 ^M (100 nM) concentration, a dose range that we and others [34] have shown to be optimal for human T cell stimulation.

Besides these Th1 and Th2 cytokines, additional cytokines were also examined including IL-12p70, IL-13 and TNF-α. Protein levels of IL-12p70, TNF-α and IFN-γ in the culture supernatants were significantly decreased, while IL-4, IL-5 and IL-13 protein levels were significantly increased by ATRA treatment in all donors tested (Table 1). ATRA and 9-cis-RA were equipotent in their effect on the majority of these cytokines. However, 9-cis-RA was significantly more effective than ATRA in inhibiting TNF-α. ATRA and 9-cis-RA failed to significantly alter IL-10 and IL-15 cytokine production by stimulated human PBMCs. While not shown, IL-18 levels were not consistently affected by ATRA or 9-cis-RA.

**Table 1:** Effects of ATRA and 9-cis-RA on human type 1 and type 2 cytokine expression by human PBMC post TCR stimulation^a^.

**Cytokine**	**Control^a^**	**ATRA^a^**	**9-*cis*-RA^a^**	**Significance^c^**
**IL-4 (pg/ml)**	36 ± 15	79 ± 9	73 ± 14	Control vs ATRA, *p *= 0.0003 Control vs 9-*cis*-RA, *p *= 0.010 ATRA vs 9-*cis*-RA, NS
**IL-5 (pg/ml)**	174 ± 39	579 ± 193	596 ± 179	Control vs ATRA, p = 0.034 Control vs 9-*cis*-RA, p = 0.019 ATRA vs 9-*cis*-RA, NS
**IL-10 (pg/ml)**	182 ± 13	172 ± 31	148 ± 31	Control vs ATRA, NS Control vs 9-*cis*-RA, NS ATRA vs 9-*cis*-RA, NS
**IL-13 (ng/ml)**	1.10 ± 0.15	1.80 ± 0.26	1.90 ± 0.25	Control vs ATRA, *p *= 0.0003 Control vs 9-*cis*-RA, *p *= 0.010 ATRA vs 9-*cis*-RA, NS
**IFN-γ (ng/ml)**	14.5 ± 3.4	7.4 ± 1.9	6.1 ± 1.5	Control vs ATRA, *p *= 0.0077 Control vs 9-*cis*-RA, *p *= 0.0065 ATRA vs 9-*cis*-RA, NS
**IL-12 (pg/ml)**	243 ± 35	61 ± 12	50 ± 10	Control vs ATRA, *p *= 0.0002 Control vs 9-*cis*-RA, *p *= 0.0003 ATRA vs 9-*cis*-RA, NS
**IL-15 (pg/ml)**	100 ± 6	111 ± 10	113 ± 7	Control vs ATRA, NS Control vs 9-*cis*-RA, NS ATRA vs 9-*cis*-RA, NS
**TNF-α (ng/ml)**	6.38 ± 1.04	2.86 ± 0.31	2.13 ± 0.36	Control vs ATRA, *p *= 0.0030 Control vs 9-*cis*-RA, *p *= 0.0006 ATRA vs 9-*cis*-RA, *p *= 0.049

^a ^Supernatants of anti-CD3 mAb(200 ng/ml)-activated human *PBMC *(2.5 × 10^6^/ml) treated with ETOH, ATRA (10^-7 ^M), or 9-cis-RA (10^-7 ^M) for 48 h were examined for the expression of various cytokines by ELISA analysis.

^b ^The results are expressed either as ng/ml or pg/ml ± SD (as designated in the cytokine column above). The data and statistical calculations were performed with data derived from at least 5 different donors.

^c ^The data was analyzed for equality of variance using Fischer's F test. If the variance was heterogeneous, the appropriate transformation of the data was performed. A two-tailed paired T test was then used to determine statistical significance. A *P *< 0.05 was considered statistically significant for all analysis.

As PBMCs are a heterogeneous population of cytokine producing cell populations, we also examined the influence of ATRA on Th1- and Th2-associated cytokine production from purified CD4^+^, CD8^+ ^and non-fractionated T cells. Similar to the above results, our data revealed that IL-4 and IL-5 synthesis was increased and IFN-γ expression was inhibited in the supernatants of all stimulated T cell populations examined (data not shown). The ATRA-induced changes in the ratio of these two cytokines were greatest in PBMCs followed by CD4^+ ^T cells, T cells and then by CD8^+ ^T cells.

ATRA also modulated the expression of several other inflammatory cytokines and chemokines by human PBMCs (Table 2). Many of these factors have not been previously shown to be influenced by retinoids. Interestingly, the expression of IL-8 and MCP-1 were both augmented by ATRA treatment, while the expression of RANTES, MIP-1β, G-CSF, IL-1β and IL-6 were significantly inhibited by ATRA treatment of anti-CD3 mAb-stimulated human PBMCs. While MIP-1α was not significantly different between the control and ATRA-treated groups, there was a trend towards downregulation (p = 0.09). IL-17, GM-CSF and IFN-α failed to demonstrate any significant differences between control and experimental cultures. The relevance of exogenous or endogenous retinoids and their nuclear receptors in the expression and production of various cytokines and chemokines remains to be defined.

**Table 2:** Effects of ATRA on human chemokine and inflammatory cytokine expression by human PBMC post TCR stimulation^a^.

**Cytokine (pg/ml)**	**Control^b^**	**ATRA^b^**	**Significance^c^**
**IL-8**	0.42 ± 0.13	0.62 ± 0.15	p = 0.002^c^
**MIP-1α**	90.1 ± 18.1	60.0 ± 14.7	NS
**RANTES**	2.1 ± 0.92	1.8 ± 0.89	p = 0.01^c^
**MCP-1**	65.4 ± 23.3	107.4 ± 29.2	p = 0.006^c^
**IL-1β**	1.7 ± 0.4	1.2 ± 0.20	p = 0.02^c^
**IL-6**	31.0 ± 7.2	13.7 ± 3.7	p = 0.007^c^
**IL-17**	1.8 ± 0.52	1.7 ± 0.56	NS
**GM-CSF**	3.4 ± 2.3	3.2 ± 2.5	NS
**G-CSF**	2.2 ± 0.76	1.2 ± 0.24	p = 0.04^c^
**IFN-α**	2.4 ± 1.1	1.4 ± 0.93	NS

^a ^Anti-CD3 antibody(200 ng/ml)-activated human PBMC (2.5 × 10^6^/ml) derived from at least 5 different donors were treated with either ETOH or ATRA (10^-7 ^M) for 48 h after which the supernatants were examined for the expression of various chemokines and cytokines by ELISA and multiplex analysis.

^b ^The results are expressed as the average cytokine value in ng/ml ± SD. The data and statistical calculations were performed with data derived from at least 5 different donors.

^c ^The data was analyzed for equality of variance using Fischer's F test. If the variance was heterogeneous, the appropriate transformation of the data was performed. A two-tailed paired T test was then used to determine statistical significance. A p < 0.05 was considered statistically significant for all analysis. NS = not significant.

We next sought to determine whether the RA-induced decrease in IFN-γ and increase in IL-4 production may be due to a shift in the frequency of cytokine producing T cells and/or the quantity of cytokine being produced by individual cytokine-producing cell. Using intracellular flow cytometric analysis of several donors, we observed a decrease in the percentage of T cells staining for IFN-γ in cultures treated with either ATRA (5.2%) or 9-cis-RA (6.3%) when compared to control T cells (15.8%). Similar studies were performed for IL-4 and IL-5; however, consistently poor intracellular staining for these cytokines was observed using several human T cell donors in our hands. So to more accurately measure the effects of RA on the frequency of Th2 cytokine production, ELISPOT analysis was performed. The results in Table 3 demonstrate the representative results of anti-CD3 mAb-activated T cells derived from single donor of two examined where a range of an approximate 3-fold increase in the frequency of IL-4- and an approximate 2-fold increase in the frequency of IL-5-producing T cells in ATRA-treated T cell cultures compared to vehicle-treated cells. In addition, a 1.5-fold decrease in IFN-γ-producing T cells was also observed between cultures treated with ATRA compared to a vehicle control. These data suggest that treatment with RA results in an increase in the frequency of T cells producing Th2 cytokines, possibly through the direct induction of type 2 cytokine or transcription factor RNA.

**Table 3:** ATRA increases the frequency of human Th2 cytokine producing T cells post TCR stimulation^a^.

**Culture Conditions**	**IL-4^b^**	**IL-5^b^**	**IFN-γ^c, d^**
**Media**	138 ± 35	322 ± 26	489 ± 57
**EtOH**	156 ± 18	312 ± 38	552 ± 82
**ATRA**	464 ± 52*	685 ± 47*	384 ± 26*

^a ^Purified human T cells (1 × 10^4^, 5 × 10^4 ^or 1 × 10^5^) were cultured in 96-well cytokine antibody-coated ELISPOT plates for 48 h in the presence or absence of anti-CD3 mAb. After incubation, the plates were washed and developed according to the manufacturer's instructions (as described in the *Methods*).

^b ^The results are expressed as the mean number of spots per 10^5 ^T cells ± SD. Based on the differences in the degree of stimulation in response to anti-CD3 mAb between the three donors examined by ELISPOT, only data from a representative donor is shown in this Table.

^c ^These ELISPOT data are in agreement with and support the intracellular cytokine flow cytometric analysis we also where we observed a decrease in the percentage of T cells derived from several donors (n > 3) staining for IFN-γ in cultures treated with either ATRA (5.2%) or 9-*cis*-RA (6.3%) when compared to control T cells (15.8%).

^d ^A two-tailed paired T test was used to determine statistical significance with * indicating p < 0.05.

### ATRA inhibits type 1 and promotes type 2 reactions even under Th1- or Th2-polarizing culture conditions

**Figure 2:**
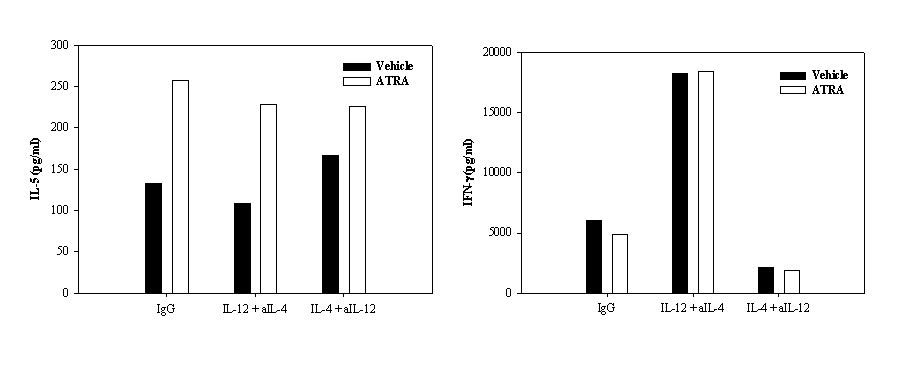
**ATRA facilitates type 2 and inhibits type 1 cytokine switching even in cultures favoring Th1 or Th2 polarization**. IL-5 and IFN-(proteins were quantitated by ELISA in supernatants of 48 h, anti-CD3 mAb (200 ng/ml)-activated PBMC (2.5 × 10^6^/ml) treated with ETOH (□) or ATRA 10^-7 ^M (■) under control (irrelevant isotype control mAb), Th1 (IL-12 and anti-IL-4 mAb) or Th2 (IL-4 and anti-IL-12) as described in the *Methods*. A representative experiment of three performed is shown and the results are expressed as pg/ml for IFN-(and IL-5. As the degree of IL-4 and IL-12 stimulation varied between donors, the data was not able to be pooled and analyzed statistically; however, all of the donors examined demonstrated similar patterns of enhancement and inhibition as shown in the current graph.

We believe that inhibition of accessory cell cytokine production by ATRA or 9-cis-RA may account for the greater type 2 polarization observed in PBMCs versus non-fractionated T cells or the CD4^+ ^and CD8^+ ^T cell populations. We therefore examined whether ATRA-induced alterations in IL-4, IL-5 and IL-13 production persisted under strong Th1- or Th2-polarizing conditions. ATRA-induced changes in IL-5 and IL-13 were not further altered upon the addition of exogenous IL-12, IL-4, or neutralizing anti-IL-4 and anti-IL-12 antibody (Figure 2). The biological activity of such manipulation is evident from corresponding increase or decrease in IFN-γ production. Due to extreme variations in the degree of stimulation by IL-4 and IL-12 in the human donors examined, only a representative experiment with one donor is shown in Figure 2. Three individual donors were examined in this experimental series and each donor demonstrated similar effects. Moreover, we have found that the addition of other potential type 1-inducing cytokines including IFN-γ or IL-18 in the presence of neutralizing anti-IL-4 antibody also failed to alter the effect of ATRA on IL-5 or IL-13 production within the cultures (data not shown). Moreover, similar to primary human T cells and these polarization cultures, we have also found that ATRA increases the proliferation and IL-4 production by human and murine Th2 clones with little to no major effects or inhibition on Th1 clones (data not shown).

### ATRA promotes Th2 responses in the presence or absence of CD28 co-stimulation

**Figure 3:**
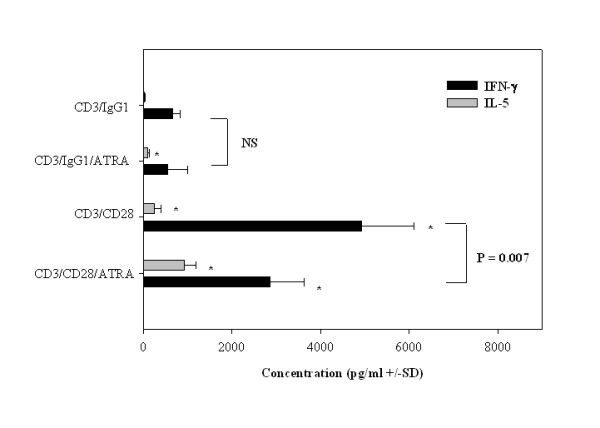
**Effects of CD28 co-stimulation on Th1 and Th2 cytokine production by ATRA-treated T cells**. IL-5 and IFN-(proteins were quantitated by ELISA in supernatants of purified T cells cultured (1 × 10^6^/ml) with immobilized anti-CD3 mAb (200 ng/ml) ± 1 ug/ml anti-CD28 mAb and treated with ETOH (□) or ATRA 10^-7 ^M (■) for 48 h. The results are expressed as pg/ml (+/- SD) for IFN-(and IL-5. A paired T test was performed on the values derived from three separate experiments as described in the *Methods*. The P values listed with the brackets compared the significant changes in IFN-γ expression between anti-CD3 mAb +/- CD28 mAb in the presence or absence of ATRA. *P < 0.05 indicates significant differences between anti-CD3 mAb + control IgG1 treated samples and the other experimental groups.

**Figure 4:**
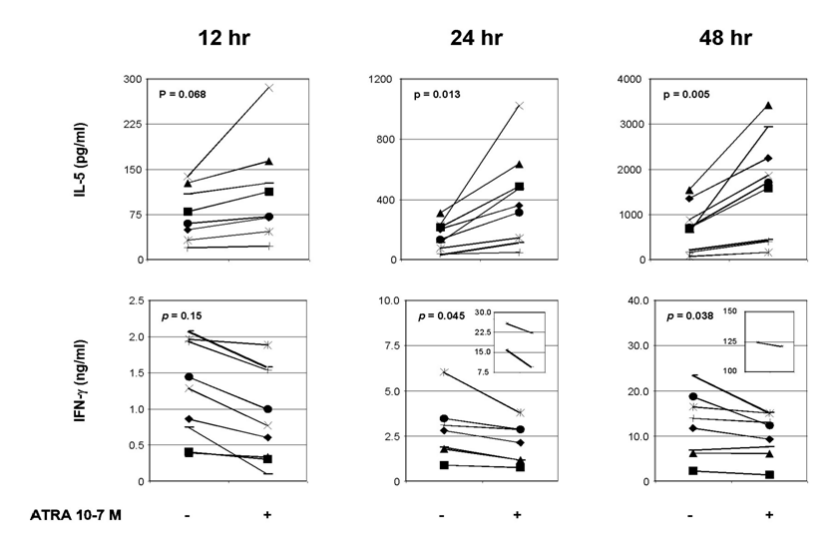
**Kinetics of ATRA-induced IL-5 production and inhibition of IFN-(synthesis post activation of CD4^+ ^T cells**. IL-5 and IFN-(proteins were quantitated by ELISA in supernatants of CD4^+ ^T cells cultured (1 × 10^6 ^ml) with immobilized anti-CD3 mAb (200 ng/ml) and anti-CD28 (1 μg/ml) and treated with ETOH (-) or ATRA 10^-7 ^M (+) for 12, 24 or 48 h. Data from 8 independent experiments is shown here. A paired T test was performed on donor T cell values from each time period as described in the *Methods*. The results are expressed as pg/ml for IL-5 and ng/ml for IFN-(.

To assess whether this same requirement was necessary for RA using human cells, purified human T cells were stimulated with anti-CD3 mAb and ATRA in the presence or absence of CD28 costimulation. The results in Figure 4 demonstrate that even in the absence of CD28 costimulation, ATRA increases IL-5 production by primary human T cells. Upon stimulation with anti-CD28mAb, there is even a greater level of IL-5 production by T cells stimulated with ATRA. In addition, CD28-costimulation was previous found to be necessary to observe the IFN-γ-inhibitory effects of ATRA using a murine model [35]. Similarly, we have found that ATRA inhibited IFN-γ production in the presence of anti-CD28 mAb (Figure 3). Thus, ATRA is capable of inducing Th2 cytokine expression in the absence of costimulatory signals such as CD28.

As type 2 differentiation is thought to involve two temporally separate signals, a differentiation and proliferation signal [36], we next examined the time course of ATRA-induced Th2 polarization of CD4^+ ^T cells in response to anti-CD3 and -CD28 mAb. As shown in Figure 4, the effects of ATRA were evident as early as 12 hrs after the initiation of the cultures. However, these differences did not reach statistical significance for IL-5 levels until 24 and 48 hr of culture. Similar trends for IL-4 expression were observed as shown here for IL-5 (data not shown).

### ATRA and 9-cis-RA induced Th2 cell polarization occurs at the mRNA level

**Figure 5:**
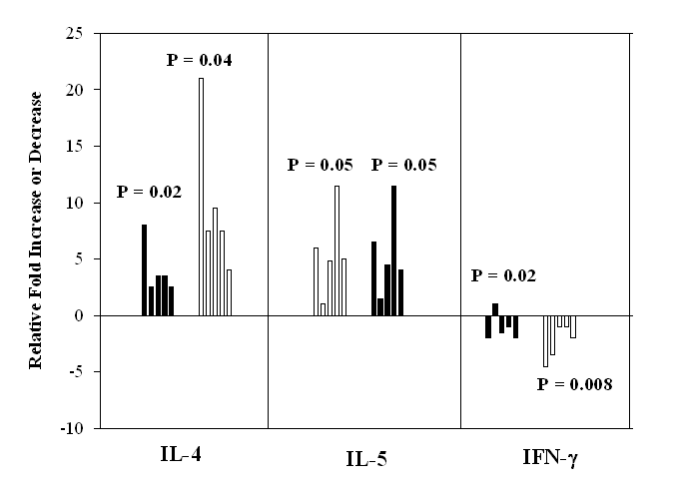
**ATRA and 9-cis-RA upregulate the expression of IL-4 and IL-5 mRNA and down-regulates the expression of IFN-(within anti-CD3 mAb-stimulated PBMC**. Taqman^® ^semi-quantitative PCR for IL-4, IL-5 and IFN-(transcripts was performed using total cellular RNA of 48 h, anti-CD3 mAb-activated PBMC (2.5 × 10^6^/ml) treated with ETOH or 10^-7 ^M of ATRA (□) or 9-*cis-*RA (■). The media used in this experimental series also contained a low dose of rhIL-2 (10 U/ml). Values obtained for each cytokine message was normalized to that obtained for 18S rRNA in the same sample as described in the *Methods*. The normalized values were then expressed as a function of the ETOH control sample. The relative copy number of PBMC IL-4 and IL-5 was increased by ATRA and 9-*cis*-RA in all donors tested, while the relative copy number of IFN-(mRNA were decreased by ATRA. The results are expressed as relative fold increase or decrease in mRNA expression. A paired T test was performed on the values derived from five donors as described in the *Methods *and the P values from the pooled donor data comparisons are listed for each cytokine and RA stimulation.

**Figure 6:**
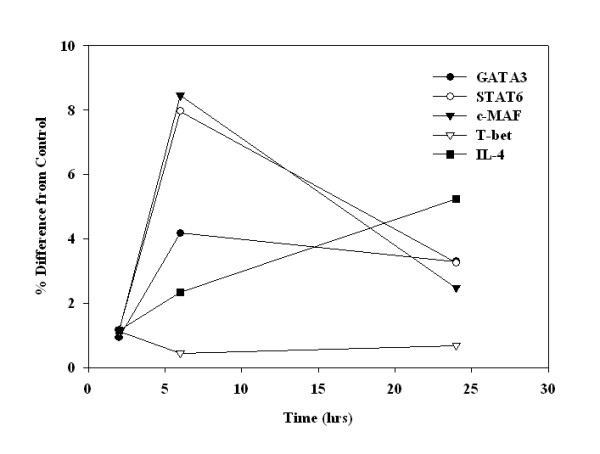
**ATRA upregulates the expression of Th2 transcription factors within anti-CD3 mAb-stimulated T cells**. Taqman^® ^semi-quantitative PCR for the Th2 transcription factors, GATA-3, STAT6 and c-MAF, and the Th1 transcription factor, T-bet, were examined using total cellular RNA derived from 2, 6 and 24 hour stimulated anti-CD3 mAb-stimulated T cells (2.5 × 10^6^/ml) treated with ETOH or ATRA (10^-7 ^M). In addition, IL-4 and GAPDH assessments were also performed. Values obtained for each cytokine message was normalized to that obtained for GAPDH in the same sample as described in the *Methods*. The normalized values were then expressed as a function of the ETOH control sample. The data shown is representative of two donors examined.

We next examined the effects of ATRA and 9-*cis*-RA on the level of mRNA of each Th1- or Th2-associated cytokine. As shown in Figure 5, the relative copy number of IL-4 and IL-5 mRNA was significantly increased by ATRA and 9-*cis*-RA utilizing PBMC derived from 5 different donors. Although similar levels of IL-4 protein were induced by both retinoids, 9-*cis*-RA was found to be at least two-fold more effective at inducing IL-4 message compared to ATRA. In addition, the relative copy number of IFN-γ mRNA was modestly but significantly decreased by both 9-*cis*-RA and ATRA. The results in Figure 5 were performed in the presence of low dose IL-2 to assist in cell activation and the maintenance of the cells. Similar results were observed in the absence of IL-2 or in the presence of anti-CD28 mAb (data not shown). Moreover, ATRA treatment of human T cells over various time intervals enhanced the expression of the Th2 cytokine, IL-4 and the Th2 transcription factors, GATA-3, c-MAF and STAT6 at 6 and 24 hours post stimulation and decreased the expression of the Th1 transcription factor, T-bet, at these same time points (Figure 6). The specific effects on the expression of Th2 transcription factors and subsequently cytokine production strongly support the ability of RA to facilitate the polarization of human T cells to a Th2 phenotype.

## Discussion

In the current study, we have described induction of the expression of the type 2 cytokines, IL-4, IL-5, and IL-13, and the inhibition of IFN-γ production upon treatment of human T cells with either ATRA or 9-*cis*-RA. To our knowledge, this is the first report describing retinoid-induced stimulation of IL-4 and IL-13 synthesis in human T cells. Furthermore, our IL-5 production data supports other reports that describe a decrease in IL-5-secreting T cells in VA-deficient mice and an increase in murine and human IL-5 synthesis by ATRA treatment of activated T cells *in vitro *[13,18]. This suggests a coordinated regulation of IL-4, IL-5, and IL-13 production from T cells [37]; however, there are important differences in the activation stimuli, kinetics, transcription factors, and cell types which govern their production. The production of IL-4, IL-5, and IL-13 is frequently, but not exclusively, co-expressed by individual CD4^+ ^T cells [38-40]. *In vivo *murine models suggest that CD28 co-stimulation is required to induce IL-4-, but not IL-13-dependent responses [41]. There are reported differences in the regulation of IL-4, IL-5, and IL-13 by transcription factors including c-MAF [42,43]. Furthermore, human CD8^+ ^cells reportedly produce low levels of IL-4 but substantial quantities of IL-5 and IL-13 [44]. The potential independent regulation of the production of these cytokines was also reflected in our observation of a limited effect of IL-4 or IL-12 addition or neutralization on IL-5 and IL-13 production.

One question that may arise from these studies is the vitamin A status of the donors utilized in these experiments. Serum for tissue culture contains a variable amount of vitamin A and a low amount of retinoic acid [45] and the individuals that the PBMC and T cells were obtained from are most likely vitamin A sufficient. The use of charcoal-stripped media to reduce the vitamin A levels used in cell culture adds the potential for artifactual data generation because there is no selectivity in the removal process. Similarly, the use of serum free media adds to the potential for artifactual data generation because it frequently provides a minimal set of nutrients (HL-1) and/or artificial growth stimulators such as diacylglycerol (AIM-V). Human T cells [46], Epstein-Barr Virus (EBV)-transformed human B cell lines [47] and myelocytic leukemia cells (HL-60) cells are known to contain esterified vitamin A [48]. This would allow the cells to survive for the 10–14 days in serum-free media it takes to deplete them of endogenous retinoids [35]. However, the time spent ex vivo greatly adds to the potential for artifactual data generation. Perhaps for these reasons, most recent studies, including many human studies on the potential immunoregulatory mechanisms of retinoids in primary T cells or PBMCs generally use unadulterated FBS and cells from vitamin A sufficient animals or humans [13,34,45,49,50]. In addition, retinoic acid is present in human plasma at a concentration of around 10–20 nM mainly in two stereoisomeric forms, all-trans-RA (tRA, ~75%) and 13-cis-RA (~25%) [51]. The concentration of RA in most rat and human tissues is higher than the plasma concentration [52,53]. When rats, mice and humans are supplemented with pharmacological amounts of RA, plasma RA concentrations can approach 0.5–3.0 uM [54]. A very large number of studies, including several studies similar to ours have used ATRA concentrations of 0.5 to 1.0 e-7M ATRA, doses that the authors had previously determined to be optimal in their systems [13,34,45,50].

Our observation that ATRA and 9-*cis*-RA had no consistent effect on IL-10 production was unexpected. A previous report indicated that overexpression of IL-10 mRNA occurs during VA deficiency in rats [56]. However, several studies have reported either a stimulatory or no effect of retinoids on IL-10 synthesis [12,15,18]. While our data strongly support a RA-induced type 2 response, the lack of a consistent effect of RA on IL-10 production provides compelling evidence that IL-10 may not be an ideal indicator of a type 2 phenotype [1]. IL-10 has not only been shown to be expressed during anti-inflammatory and type 2 responses [1], but also has been reported to be expressed by CD4^+ ^T cells and T cell clones producing both IFN-γ and IL-10 [1].

A role for retinoids in inhibiting IL-12 synthesis in humans is consistent with data from studies describing overexpression of IL-12p40 mRNA during VA deficiency in mice [17] and rats [56] as well as from several *in vitro *studies demonstrating diverse inhibitory effects of retinoids on IL-12 production by LPS- or KLH-stimulated mouse macrophages [33,57]. However, our current studies suggest that ATRA-induced reduction in IL-12 expression is not a critical factor in RA-induced type 2 polarization of human T cells. Furthermore, in contrast to our current findings, the addition of exogenous IL-12 to cultures reduced ATRA and 9-*cis*-RA-induced murine T cell IL-4 synthesis to the level of control cultures [33] and the neutralization of IL-4 within the cultures decreased the type 2-promoting effect of 9-*cis*-RA [26]. Our current data also contrast with the recent studies suggesting that the RA-induced type 2 polarization of T cells is exclusively regulated at the level of the APC [28,33]. In these studies, RA-pretreated, LPS-stimulated macrophages induced antigen-specific T cells to polarize into Th2-like cells in the absence of exogenous RA, presumably through reduced IL-12 synthesis. However, our studies demonstrate an IL-12 independent role for ATRA in type 2 cytokine polarization *in vitro*.

Data presented herein showing the equipotency of ATRA and 9-*cis*-RA in promoting a type 2 response in human T cells contrasts with *in vitro *murine T cell development studies where 9-*cis*-RA, but not ATRA, stimulated Th2 development [58]. Our data suggest the involvement of ligated nuclear retinoic acid receptors (RARs) but not retinoid X receptors (RXRs) in Th2 differentiation as ATRA binds to only to RARs and 9-*cis*-RA binds to both RARs and RXRs. We have found that RARα agonists recapitulate the effect of retinoic acid shown here and the use of a RARα antagonist inhibits the effect of retinoic acid on the Th2-related responses (data not shown). These data must be interpreted with caution because of the non-specific interconversion of ATRA and 9-*cis*-RA under normal cell culture conditions [59]. In addition, we can not rule out the possibility that RXR receptors also play a role in Th2 development as have recently been shown in murine T cells [60]. We are currently exploring which nuclear receptors (RAR and/or RXR) are involved in ATRA-induced Th2 differentiation by the use of stable receptor-selective retinoids.

Costimulation with CD28 reportedly favors development of human type 2 T cells *in vitro *[61]. CD28-costimulation was also necessary to observe the IFN-γ-inhibitory effects of ATRA using a murine model [36]. However, our observation that the preservation of the ATRA-induced type 2 responses by T cells in the absence of CD28-costimulation is unique. The differential CD28-dependency of these two responses is unknown but is supported by previous data which suggests that CD28 costimulation is required for inhibition of IFN-γ production but not induction of IL-5 production during Th2 polarization [62]. Unlike murine T cells [35], we have also observed a relatively equal decrease (ranging from 10–30%) in the frequency (Table 3) and the percentage and intensity of T cells staining for IFN-γ (intracellular flow analysis, data not shown) in cultures treated with ATRA and 9-*cis*-RA in the presence or absence of CD28 mAb. These data are inconsistent with a murine study where ATRA failed to alter the frequency of the IFN-γ secreting T cells [18]. Our preliminary observations demonstrating that this decrease appears to only occur within TNF-α/IFN-γ double producing T cells supports a general negative regulatory effect of retinoids on type 1 cytokine production. These cytokines have been reported to be frequently co-expressed by pathogen-generated human Th1 clones [40].

What are the potential mechanisms for direct induction of Th2 differentiation by ATRA or 9-*cis*-RA? It is possible that RA directly activates transcription through its nuclear receptors, RAR and or RXRs; however, our search of the 5' promoter region of the Th2 cytokine genes, IL-4, IL-5 and IL-13, did not reveal any RAR or RXR-response elements. The refractory nature of retinoid-induced Th2 differentiation to changes in IL-4 and IL-12 levels and CD28-mediated costimulation suggests that ATRA may act through a master Th2 differentiation factor such as Ets-1, cMAF, GATA-3 or STAT-6. This is consistent with recent data obtained from tumor cell lines where RA induced the synthesis of Ets-1 [63] and STAT-6 [64], transcription factors that are involved in Th2 differentiation [2]. Ectopic expression of GATA-3 in Th1 cells induces IL-4, IL-5, and IL-13 production and ectopic expression of Stat6 in Th1 cells induces the production of Th2-cytokine such as IL-4 and IL-5 [65]. STAT-6 also induces the expression of Ets-1, MAF and GATA-3 and reduces the expression of IL-12R∃2 message independently of IL-4 production [65]. Here, through the use of real time RT-PCR using several of the retinoid-treated T cells and PBMCs, we also observed increases in the gene expression of the Th2 factors, cMAF, GATA-3, and STAT-6, using ATRA and a concomitant decrease in the expression of the Th1 factor, T-bet, 4–12 hours post stimulation (Figure 6). These data strongly support a specific role for retinoids in the development of Th2 cells.

## Conclusion

In conclusion, we have demonstrated that ATRA and 9-*cis*-RA increase the expression of IL-4, IL-5 and IL-13 but not IL-10 mRNA and protein from activated human T cells. ATRA acts early and directly polarizes T cells towards type 2 responses even in the presence of type 1-inducing signals or in the absence of CD28-costimulation. Although ATRA decreased IL-12 synthesis within PBMC cultures, this was not obligatory as RA directly induced type 2 cytokine production by highly purified human T cells in the absence of APCs. A better understanding of the type 2 cytokine promoting activity of ATRA and 9-*cis*-RA in human T cells would provide better clinical interventions to boost vaccine efficacy to certain antigens or to reduce various types of pro-inflammatory and autoimmune pathologies [66].

## Methods

### Reagents

ATRA, 9-*cis*-RA were purchased from Sigma, St. Louis, MO. Retinoids were dissolved at various concentrations in 100% ETOH, overlayered with argon gas and stored at -80°C in the dark until used. Recombinant human IFN-γ, IL-4, IL-12 and IL-18 were obtained from R & D Systems (Minneapolis, MN). Recombinant IFN-γ was obtained from Biosource Int. (Camarillo, CA). Neutralizing monoclonal antibodies (mAbs) to human IFN-γ (clone 25718.111) IL-4 (clone 34019.111), and IL-12 (clone 24910.1) were also obtained from R & D Systems. Based upon manufacturer's testing lot-specific testing, 1 :g of anti-IFN-γ, anti-IL-4, and anti-IL-12 will neutralize 5 ng/ml, 300 pg/ml and 700 pg/ml of rhIFN-γ, rhIL-4, and rhIL-12, respectively.

### Cell preparation

Whole blood was acquired from healthy human volunteers between the ages of 21–55 years who provided informed consent. PBMC were isolated by Ficoll Paque (Amersham Pharmacia Biotech, Piscataway, NJ) density gradient centrifugation followed by treatment with ammonium chloride (ACK) lysis solution (Biofluids, Gaithersburg, MD) to eliminate the remaining erythrocytes. The isolated cells were subsequently washed 2 times in PBS and resuspended in RPMI 1640 (Biofluids) supplemented with 10% heat-inactivated FBS (Sigma), 2% heat-inactivated pooled human AB serum (Sigma), 50 μM mercaptoethanol (Gibco BRL Gaithersburg, MD), 1 mM sodium pyruvate (Biofluids), 2 mM glutamine, 1 × non-essential amino acid solution (Biofluids), 1 mg/ml gentamicin (Biowhittaker, Walkersville, MD), 100 U/ml penicillin (Biofluids), 100 :g/ml streptomycin (Biofluids), and 20 mM HEPES buffer (Biofluids). T cells, CD4^+ ^T cells and CD8^+ ^T cells were isolated by negative selection using enrichment columns according to manufacturer's instructions (R & D Systems). All of these cells were typically > 93% pure as assessed by flow cytometric analysis. The contaminating cell population was largely CD8^+ ^cells. Most likely, these cells were NK cells based on their size and granularity.

### Cell culture and harvest

PBMC (2.5 × 10 ^6 ^cells/ml) were activated with 200 ng/ml of immobilized anti-CD3ε OKT-3, Ortho, Raritan, NJ) ± 0.001 to 1 μM of various retinoids or ETOH vehicle control for 48 h. IL-2 (Teceleukin, Hoffman LaRoche, Nutley, NJ) at 10 U/ml was added where indicated. Where indicated, 1 μg/ml of neutralizing anti-cytokine mAb was added to the cultures. Alternatively, T cells (1.0 × 10 ^6 ^cells/ml) were activated with 200 ng/ml of immobilized anti-CD3 and 0.1 to 1 μg of soluble anti-CD28 (clone 28.2, Pharmingen) or IL-2 at 10 U/ml was added where indicated to provide co-activation or costimulatory signals. PBMCs or T cells were harvested at various times after incubation at 37° and 5% CO_2_. The 48 hr time interval was selected for many of the studies shown based on the optimal and reproducible cytokine expression in response to anti-CD3 mAb. Non-adherent cells were decanted from the flasks and centrifuged to obtain supernatants. The flasks were then treated with Enzyme-Free cell disassociation solution (Specialty Media, Phillipsburg, NJ) to remove the adherent cells (a typical result of cell activation and the anti-CD3 coated flasks) and were gently scraped to remove and harvest the cells. Viable cells from the decanted cells and cell removal mixture were isolated by Ficoll Paque density gradient centrifugation (as described above). Cell viability was not significantly affected by this enzyme treatment process (viability >95%).

It should be noted that we have also utilized the serum free medium AIMV in these various cultures and observed similar effects to serum containing medium (data not shown).

### Cytokine ELISA

ELISA (Biosource) and Bio-Plex Human Cytokine 17-Plex (Biorad, Hercules, CA) were utilized to examine the following human-specific cytokines: IFN-α, IFN-γ, IL-1α, IL-1β, IL-4, IL-5, IL-6, IL-7, IL-8, IL-10, IL-12 p70, IL-13, IL-15, IL-17, G-CSF, GM-CSF, MIP-1∀, MIP-1∃, RANTES, MCP-1 and TNF-α. The IL-18 ELISA was obtained from R & D Systems (Minneapolis, MN). All of the ELISAs and multiplex assays were performed according to the manufacturer's instructions. The results are expressed as pg/ml or ng/ml and all assays were run in duplicate with at least three separate experiments being examined.

### Real Time PCR

Cytoplasmic RNA was extracted and purified using a commercially available kit (RNAeasy, Qiagen, Valencia, CA). Purified RNA was electrophoresed on a 1% agarose gel to assess the integrity of the purified RNA. One :g of RNA was reverse transcribed into cDNA using a commercial available kit (Applied Biosystems, Foster City, CA). One hundred pg RNA equivalent of this cDNA was used for PCR amplification. PCR reactions were performed in special optical tubes in a 96 well microtiter plate format on an ABI PRISM 7700 Sequence Detector System (PE Applied Biosystems) using pre-developed FAM- and TAMRA-labeled internal oligonucleotide probes and primers for IFN-γ, IL-4, IL-5, IL-10, IL-12p30, IL-12p40, IL-15, and TNF-α (PE Applied Biosystems). Each reagent also contains VIC- and TAMRA-labeled internal oligonucleotide probes and primers specific for the 18S RNA ribosomal subunit. Amplification conditions were as follows 25°C for two min; 95°C for 10 min; 40 cycles of 95°C 15 s and 60°C for 1 min. Fluorescence signals measured during amplification were processed post-amplification and were regarded as positive if the fluorescence intensity was ten fold greater than the standard deviation of the baseline fluorescence. This level is defined as the threshold cycle (Ct). The Ct value for 18S ribosomal subunit was subtracted from the Ct value for each cytokine message to normalize for RNA content. This value is defined as ΔCT. To evaluate the effects of retinoids, ΔCT_treatment _was subtracted from ΔCt_control_. This value is defined as ΔΔCT. The relative folds increase or decrease was then calculated as 2 ^-ΔΔCT^.

The PCR was also set up using SYBR green Master Mix (Applied Biosystems), 1 μl cDNA and gene-specific primers at a final concentration of 0.3 μM. Thermal cycling was carried out on the Applied Biosystems GeneAmp 7700 Sequence Detector and SYBR green dye intensity was analyzed using GeneAmp 7700 SDS software. Primers for human GATA3, STAT6, C-MAF, and T-bet genes and glyceraldehyde-3-phosphate dehydrogenase (GAPDH) as control were designed using ABI prism software (PE Applied Biosystems). Primers are available upon request. Similar amplification procedures and data computation were followed as described above. No PCR products were generated from genomic *versus *cDNA template.

### Intracellular Flow Cytometry and ELISPOT analysis

Mouse anti-human CD3-Cy-Chrome^®^, anti-human IL-4-phycoerytrin (PE), anti-human IFN-(-fluorescein isothiocyanate (FITC) anti-human TNF-∀-FITC, and the appropriately labeled isotype control mAbs were obtained from Pharmingen. PBMCs were cultured for 36 hrs as described above after which the cells were treated with 2 :M monensin (Sigma) and subsequently harvested as described above. Cells (0.25 × 10^6^) were suspended in 50 :L of staining buffer (1% FCS, 1% goat serum, 2.5 :g of mouse IgG/50 μL) in round-bottom 96-well plates at 4°C for 15 min. 5 :L of the appropriate dilution of each antibody was then added. Cells were then incubated for 30–40 min at 4°C. Cells were pelleted and medium was aspirated carefully. Cells were then washed with 100 :L of PBS/FBS buffer twice. Cells were fixed and permeabilized with Cytofix/Cytoperm^® ^solution (Pharmingen). Various combinations of labeled anti-human cytokines were used to stain cells. Cytochrome-conjugated mouse IgG_1 _mAb and PE mouse-IgG were used as isotype controls at the same concentrations as the anti-cytokine antibody. Additional controls in which the labeled mAbs and 10 fold saturating recombinant cytokine proteins were pre-incubated for 30 min at room temperature before staining (IL-4). Alternatively, cells were pretreated with unlabeled mAb (IFN-γ). Three-color cytofluorometry was performed using a FacScan (Becton Dickinson, San Diego, CA). A minimum of 10,000 CD3^+ ^cells were analyzed in these experiments. Data are expressed as the % of CD3^+ ^cells expressing the marker of interest or the mean channel number (MCN) of the marker's fluorescent intensity.

The ELISPOT assays (BD Biosciences) used to quantify IFN-γ-, IL-4, and IL-5-producing T cells were performed according to the manufacturer's instructions. Briefly, T cells were prepared at different cell densities ranging from 1 × 10^5^, 1 × 10^6^, and 2.5 × 10^6 ^cells per ml and 100 :l of the suspensions were added to each well of mouse anti-human cytokine antibody-coated BD ELISPOT plates. The cells were stimulated using ATRA or 9-*cis*-RA and/or soluble anti-CD3 and CD28 antibodies described above under cell culture. The plates were then incubated at 37°C in a 5% CO2 humidified incubator for 24 hr after which the cell suspension were aspirated, the wells were washed with various combinations of deionized water and wash buffer, and subsequently developed using the proper antibody, conjugate and substrate pairs defined by the manufacturer. The plates were air-dried overnight at room temperature and the plates were stored in a sealed bag in the dark until analyzed. Spots were then enumerated using an ImmunoSpot^® ^Series 2 Analyzer (Cellular Technology Limited, Cleveland, Ohio) and the supporting ImmunoSpot^® ^Software. Spots were counted by an automated system using a defined set of parameters for size, intensity, and gradient. The background (the mean numbers of spots in wells without stimulation) was subtracted from each well on each cytokine plate. A response was considered positive if the average number of cytokine-producing cells (CPC) per triplicate wells exceeded background +/- 2SD. The data are shown as the average number of CPC per 10^6 ^cells.

### Statistical analysis

Data were analyzed for equality of variance using Fischer's F test (Statview 5.0 for Macintosh, Abacus Concepts, Berkeley, CA). If the variance was heterogeneous, the appropriate transformation of the data was performed. A two-tailed paired T test was then used to determine statistical significance. A *P *< 0.05 was considered statistically significant for all analysis.

## Abbreviations

ATRA, all-*trans *retinoic acid; 9-*cis*-RA, 9-*cis-*retinoic acid; DTH, delayed type hypersensitivity; pTh, precursor T helper; RARs, retinoic acid receptors; RXRs, retinoid X receptors; VA, Vitamin A

## Authors' contributions

HD, GC, RP, MLK, AW, VDD, CN and DDT did the experiments. HD and DDT prepared the figures and co-wrote the paper. DDT supervised the work and edited the manuscript. All authors have read and approved the final version of the manuscript.
